# Milk and Dairy Products and Their Impact on Carbohydrate Metabolism and Fertility—A Potential Role in the Diet of Women with Polycystic Ovary Syndrome

**DOI:** 10.3390/nu12113491

**Published:** 2020-11-13

**Authors:** Justyna Janiszewska, Joanna Ostrowska, Dorota Szostak-Węgierek

**Affiliations:** Department of Clinical Dietetics, Faculty of Health Sciences, Medical University of Warsaw, E Ciołka Str. 27, 01-445 Warsaw, Poland; jjaniszewska@wum.edu.pl (J.J.); dorota.szostak-wegierek@wum.edu.pl (D.S.-W.)

**Keywords:** milk, dairy products, type 2 diabetes mellitus, insulin resistance, polycystic ovary syndrome, fertility, ovulation

## Abstract

Milk and dairy products are considered an important component of healthy and balanced diet and are deemed to exert a positive effect on human health. They appear to play a role in the prevention and treatment of carbohydrate balance disturbances. The products include numerous valuable components with a potential hypoglycemic activity, such as calcium, vitamin D, magnesium and probiotics. Multiple authors suggested that the consumption of dairy products was negatively associated with the risk of type 2 diabetes mellitus, insulin resistance and ovulation disorders. However, there are still numerous ambiguities concerning both the presumed protective role of dairy products in carbohydrate metabolism disorders, and the advantage of consuming low-fat dairy products over high-fat ones, especially in women with the risk of ovulation disorders. Therefore, this literature review aims at the presentation of the current state of knowledge concerning the relationship between dairy product consumption and the risk of insulin resistance, type 2 diabetes mellitus in women, and the potential effect on the course of polycystic ovary syndrome.

## 1. Introduction

Milk and dairy products have been considered as an important component of healthy and balanced diet for many years. According to Polish recommendations of the Food and Nutrition Institute [1], they should be included in everyday diet regardless of age. It is recommended that adults consume at least two glasses of milk daily. They may be replaced with yoghurt, kefir and, partially, cheese.

Cow milk contains 87% of water, 3–4% of lipids, 3.5% of protein, 5% of lactose and 1.2% of vitamins (B2, B12, A, D) and minerals (calcium, phosphorus, potassium, magnesium, zinc and selenium). Cow milk fat consists of 60% of saturated fatty acids, including mainly palmitic acid. The milk of ruminants also contains conjugated dienes of linoleic acids (CLAs) which present numerous health-promoting properties. However, the particularly nutritious value of milk is mostly due to high-quality protein which includes the whole set of exogenous amino acids necessary for the synthesis of body protein. Milk protein consists of 80% of casein, 20% of whey, which plays a role in short- and long-lasting regulation of food consumption via the induction of satiety signals, thereby promoting the maintenance of appropriate body weight. Bioactive milk peptides may exert a positive influence on human health through the regulation of physiological functions, a direct effect on metabolism and on some receptors. It was suggested that they presented antineoplastic, antihypertensive, antithrombotic and immunomodulatory properties. Milk and dairy products were also attributed favorable properties in the prevention and treatment of carbohydrate metabolism disorders [2,3,4]. Polycystic ovary syndrome (PCOS) is one of the most common endocrine disorders in women of reproductive age. It is accompanied by oligoovulation and/or the lack of ovulation, clinical and/or biochemical hyperandrogenism and the presence of polycystic ovaries in ultrasound examination [5]. It is estimated that even 90–95% of ovulatory infertility cases are caused by this medical condition. Due to the presence of endocrine and metabolic disorders, women with PCOS are a group that is particularly susceptible to the development of insulin resistance, secondary disorders of glucose tolerance and type 2 diabetes mellitus (T2DM), cardiovascular diseases and dyslipidemia [6]. Increasing attention has recently been paid to the significance of dairy products in the diet of women with PCOS, particularly comprising their influence on ovulation and fertility and the associated risk of carbohydrate metabolism disorders, such as insulin resistance or T2DM. The obtained results are frequently contradictory. Therefore, it is necessary to conduct a comprehensive overview of the most recent studies in this area.

## 2. Dairy Products and Insulin Resistance

The effect of milk and dairy products on carbohydrate metabolism is the subject of numerous studies. However, the results are still contradictory. It is known that protein consumption has the same capacity to stimulate insulin secretion as carbohydrate consumption. However, it was demonstrated that not all protein-containing products exerted the same effect on insulin secretion and modulated insulin sensitivity in tissues in various ways. Milk proteins exerted the strongest influence on the secretion of insulin and incretins compared to other animal proteins [7]. It is mostly attributed to the high content of branched-chain amino acids (leucines, isoleucines, valines) which activate various pathways associated with insulin resistance [8]. However, apart from protein components, such as insulinogenic amino acids and bioactive peptides, dairy products also contain calcium, magnesium, potassium and carbohydrates with low glycemic index, which all seem to have a favorable effect on the control of glycemia, insulin secretion, insulin sensitivity of tissues and the reduction in the risk of T2DM. Moreover, unsaturated trans fatty acids which naturally occur in milk fat modulate the expression of PPAR-γ (peroxisome proliferator-activated receptor γ) and PPAR-α (peroxisome proliferator-activated receptor α), which is also beneficial in glucose homeostasis. Furthermore, fermentation and enhancing dairy products with probiotics and vitamin D may improve their glucoregulatory activity [7,9].

According to some studies conducted in women and men, the consumption of milk and dairy products might be associated with higher tissue sensitivity to the activity of insulin and lower fasting insulin levels [10,11]. The observation was confirmed by a meta-analysis of 30 randomized clinical trials. It demonstrated that the consumption of dairy products, especially low-fat ones, was beneficial in terms of tissue insulin sensitivity [12]. However, the results of some studies suggested that only long-lasting consumption of dairy products might have a beneficial effect on insulin sensitivity in tissues. A systematic review of 10 interventional studies [13] was conducted to analyze the effect of dairy products consumption on insulin sensitivity in individuals without T2DM. It was demonstrated that improved sensitivity to insulin occurred only after 12 weeks of a diet higher in dairy content, while studies lasting below 8 weeks did not show any significant changes concerning insulin sensitivity in tissues. Similar results were obtained by Rideout et al. [14], who noticed that the values of HOMA-IR (Homeostatic Model Assessment-Insulin Resistance) markedly improved over 6 months in individuals who consumed higher amounts of low-fat dairy products (four servings of milk of yoghurt daily) compared to those who consumed less (less than two servings of milk or yoghurt daily). However, not all studies confirmed those observations. A systematic review and a meta-analysis of 44 randomized studies revealed that the increased supply of dairy products exerted no effect on fasting insulin concentrations and HOMA-IR index values in healthy diabetes-free individuals [15]. A randomized clinical trial conducted by O’Connor et al. [16] also showed no significant changes in insulin secretion and insulin sensitivity in adults with hyperinsulinemia who were characterized by high dairy consumption (>4 servings/day) compared to those who consumed small amounts of dairy (≤2 servings/day). Interesting results were obtained by Eelderink et al. [17] who demonstrated that postprandial insulin concentrations in persons consuming a diet with low dairy content (≤1 serving of dairy per day) were not significantly different compared to participants who consumed high amounts of dairy products (five servings/day in women and six servings/day in men). However, significantly higher fasting insulin and HOMA-IR values were associated with a diet high in dairy products compared to low-dairy diet (2.21 ± 0.91 versus 1.99 ± 0.72; *p* = 0.027).

There is paucity of data regarding the correlation between the consumption of milk products and the risk of insulin resistance in women. However, it may be presumed that such products may increase the risk of insulin resistance in women at various ages. Lawlor et al. [18], who investigated the association between milk consumption, insulin resistance and metabolic syndrome in 4024 British women, observed that women who had never drunk milk had lower HOMA-IR values and developed metabolic syndrome less frequently than women who regularly drank milk. Similarly, a study conducted by Tucker et al. [19] showed that the values of HOMA-IR went up with increased milk consumption in the studied women. Factors such as age, body weight, adipose tissue amount or physical activity had no significant influence on the relationship between milk consumption and HOMA-IR values. The results underlay the conclusion that long-lasting hyperinsulinemia which occurred due to high dairy consumption may be a significant predictor of insulin resistance in women. Moreover, an 8-week interventional study by Phy et al. [20] demonstrated that diet low in starch and milk products resulted in an increased sensitivity to insulin (HOMA-IR reduction by 1.9 ± 1.2, *p* < 0.001), lowered fasting insulin level (−17.0 ± 13.6 μg/mL, *p* < 0.001) and a 75 g 2 h oral glucose tolerance test (−82.8 ± 177.7 μg/mL, *p* = 0.03) in women with PCOS. An unfavorable influence of milk products in women was also confirmed in a study by von Post-Skagegård et al. [21], who demonstrated that the 120 min ratios of insulin to glucose and insulin to peptide C were significantly higher after a meal containing milk proteins compared to a meal containing fish or soy protein. Furthermore, Turner et al. [22] noted that HOMA-IR was markedly lower in women who had consumed a diet including red meat compared to diet containing milk products. Moreover, women who consumed <1 portion of milk products daily were characterized by significantly lower fasting insulin levels and HOMA-IR compared to women whose diet included from four to six portions of low-fat milk products daily.

However, not all studies indicated a negative impact of dairy product consumption on the risk of insulin resistance in women. According to some authors, the influence of dairy products was neutral or even favorable in terms of sensitivity to insulin in women. A study by Drouin-Chartier et al. [23] revealed that a diet including milk had no effect on HOMA-IR and fasting insulin levels in postmenopausal women. The Coronary Artery Risk Development in Young Adults Study (The CARDIA Study) [24] showed that a daily increase in the consumption of milk products translated into the reduction in the risk of developing insulin resistance by 30% in Black women (Odds Ratio (OR) 0.70, 95% Confidence Interval (CI), 0.54–0.91, *p* < 0.05) and by 38% in White women (OR 0.62, 95% CI, 0.46–0.84, *p* < 0.05). Yoghurt appears to be particularly beneficial in the prophylaxis of insulin resistance in women. A study by Chen et al. [25] revealed that full-fat yoghurt significantly reduced HOMA-IR, fasting insulin levels and a 75 g 2 h oral glucose tolerance test compared to full-fat milk in women with metabolic syndrome and nonalcoholic fatty liver disease. However, the study showed significantly reduced HOMA-IR, fasting glucose and insulin levels in a group of women consuming milk, while the level of insulin in a 75 g 2 h oral glucose tolerance test significantly increased. Based on the results, the authors suggested that the unfavorable influence of milk consumption on carbohydrate metabolism was not associated with weakened insulin sensitivity, but only with the fact that milk might prolong postprandial insulin secretion.

Some authors pointed out particularly beneficial properties of probiotics. A randomized clinical trial conducted in a group of women with PCOS showed that supplementation with probiotics contributed to a considerable reduction in fasting glucose levels [26]. Other studies conducted in women showed that the consumption of yoghurts fortified both with vitamin D and probiotic bacteria was associated with a significantly higher reduction in HOMA-IR and fasting insulin compared to women consuming traditional low-fat yoghurt [27,28], so the favorable properties of yoghurt may be enhanced with the addition of probiotic bacteria and vitamin D. Therefore, the consumption of yoghurt (especially the fortified types) by women seems to have a beneficial effect on tissue insulin sensitivity. However, their positive properties may not be fully confirmed due to the paucity of studies in women with PCOS. Detailed results of studies on the effects of dairy consumption on insulin resistance in women are described in Table 1.

Basing on the observations described above, it cannot be clearly determined whether the consumption of milk and dairy products has a beneficial effect on insulin sensitivity in tissues in women, and due to the lack of studies conducted in women with PCOS, it is even more difficult to draw conclusions concerning their beneficial effect in this condition. It may even be assumed that the consumption of diet with a high dairy content may be a predictor of hyperinsulinemia and insulin resistance in women. However, it is worth emphasizing that the results of some studies suggested that only long-lasting consumption of dairy products might have a beneficial effect on reducing insulin resistance in tissues. Therefore, it is necessary to conduct more well-planned randomized clinical trials in women with PCOS to provide a clear answer concerning the significance of dairy product consumption in the prevention and treatment of insulin resistance in this condition.

## 3. Dairy Products and Type 2 Diabetes Mellitus

As mentioned above, dairy products, due to their high content of whey proteins which are rich in branched-chain amino acids (leucine, isoleucine, valine) and lysine, may stimulate insulin secretion and reduce postprandial glycemia, which is particularly favorable in the prophylaxis of T2DM [8]. Conversely, the excessive amount of branched-chain amino acids in the diet is considered to lead to insulin resistance in tissues via the activation of mTOR (mammalian target of rapamycin) kinase, thereby increasing the risk of T2DM [29]. It was corroborated by a prospective study conducted in a cohort of Chinese women. The study showed that higher branched-chain amino acid content consumed with meat and dairy products in the second part of pregnancy was associated with the increase in the risk of gestational diabetes mellitus by approximately 95% [30]. According to some authors, whey proteins, by modifying gene expression, may affect glucose metabolism, also by its increased use in the liver. Moreover, the influence of dairy products on glucose metabolism and the risk of T2DM may depend on glucokinase genetic polymorphism which is specific for a particular person. Therefore, it is suggested that some individuals may find high dairy intake more beneficial than others [31]. Furthermore, it seems that hyperinsulinemia due to dairy intake may be favorable in glucose homeostasis regulation in patients with hyperglycemia and T2DM [32]. Systematic reviews and meta-analyses of observational and cohort studies in women and men [32,33,34,35,36] indicated that dairy product consumption was negatively associated with the risk of T2DM. Moreover, such a relationship was particularly intensified in cases of low-fat and fermented dairy products. A randomized study by Díaz-López et al. [37] also revealed a negative relationship between total dairy intake and the risk of T2DM. It was particularly visible in the case of low-fat dairy products. It is consistent with the results of a meta-analysis of 13 cohort studies. The meta-analysis revealed that increasing the consumption of low-fat dairy products by 200 g daily was linked to T2DM risk reduction by 4% (Relative Risk (RR) 0.96; 95% CI 0.92, 1.00; *p* = 0.072). In the case of full-fat dairy products no such correlation was observed (RR 0.98; 95% CI 0.93, 1.04; *p* = 0.52) [32]. Similar outcomes were obtained in the Lifelines Cohort Study [38], in which a 2% reduction in the risk of prediabetes was achieved with the intake of skimmed dairy products (RR 0.98; 95% CI 0.97, 1.00; *p* = 0.02) and fermented dairy products (RR 0.98; 95% CI 0.97, 0.99; *p* = 0.004) increased by 100 g daily. Conversely, the consumption of full-fat dairy products was associated with the increased risk (RR 1.03; 95% CI 1.01, 1.06; *p* = 0.004). A systematic review of meta-analyses by Drouin-Chartier et al. [39] revealed that current evidence obtained from scientific research indicated favorable or neutral interrelations between the consumption of dairy products and T2DM occurrence. However, recommendations concerning the advantage of low-fat product consumption over full-fat ones were confirmed by a low number of reliable scientific papers. Similar conclusions were reached by Yakoob et al. [40] and Guo et al. [41] indicating no convincing evidence to confirm the hypothesis stating that low-fat dairy intake was more effective in reducing the risk of type 2 diabetes compared to full-fat dairy products. Moreover, a systematic review of studies concerning the relationship between dairy product intake and the risk of cardiovascular disease showed that the consumption of full-fat, semi-skimmed and fermented dairy products was neutrally associated with the risk of T2DM, while the consumption of low-fat dairy was positively associated with the risk [36]. According to some authors, full-fat dairy products, despite the high content of saturated fatty acids, had a positive effect on human health. It was also stated that there was insufficient evidence to confirm that those fatty acids increased the risk of cardiometabolic pathologies, such as T2DM, and they might even present some protective properties [42,43,44]. It is consistent with the results of a cohort study by Korat et al. [45] who demonstrated no relationship between milk fat and the risk of T2DM both in the population of men and women.

It is considered that sex is one of the biological factors modulating the course and incidence of cardiometabolic diseases, including T2DM. An increasing amount of evidence confirmed the role of sex in the course and treatment of T2DM and its influence on the increased risk of the disease [46]. Research conducted in the populations of women indicated that a diet rich in milk products was associated with a lower risk of developing T2DM [47,48]. The observations were confirmed by systematic reviews and meta-analyses of observational and cohort studies [32,34,35], which indicated that milk product consumption was inversely correlated with the risk of T2DM in women. Moreover, Kirri et al. [49] observed that the beneficial correlation between milk product consumption and the risk of T2DM was statistically significant only in women. A prospective study by Liu et al. [50] showed that the risk of T2DM in women from the highest quintile of milk product consumption (>2.9 servings/day) was lower by 20% compared to women from the lowest quintile (<0.85 servings/day). Furthermore, each increment of the daily consumption by one serving was associated with T2DM risk reduction by 4% (RR 0.96, 95% CI, 0.93–1.0, *p* < 0.05). The beneficial effect of milk product consumption on the risk of T2DM in women is mainly attributed to low-fat milk products, while their high-fat equivalents may even increase the risk. It was confirmed by the results of a study by Margolis et al. [51] who demonstrated that the risk of T2DM in women from the highest quintile of low-fat milk product consumption was lower by 30% compared to women from the lowest quintile (RR 0.70, 95% CI, 0.64–0.77, *p* < 0.0001). However, no such correlation was demonstrated for high-fat milk products. A prospective cohort The Black Women’s Health Study [52] also showed that the consumption of low-fat milk products was associated with the risk of T2DM lower by 13% in Black women. At the same time, no such correlation was observed for high-fat milk products. Another prospective The Nurses’ Health Study [53] revealed a 25% lower risk of T2DM (RR 0.75; 95% CI 0.55, 1.02, *p* = 0.03) in women from the highest quintile of total milk product consumption compared to women from the lowest quintile. The beneficial influence of milk product consumption was observed both in the cases of low-fat and high-fat products. Moreover, constant high consumption of milk products continued in adulthood was also associated with a lower risk of T2DM, which might suggest that long-lasting milk product consumption might be beneficial in the context of the prophylaxis of T2DM in women.

Yoghurt appears to be a particularly important dairy product. It should be introduced into the diet of women with PCOS because of strong scientific evidence suggestive of the relationship between its consumption and lowering the risk of developing type 2 diabetes. Yoghurt consumption increases the concentrations of circulating anorexic peptides—glucagon-like peptide 1 (GLP-1) and peptide YY (PYY), whose activity is associated with the improvement of glucose homeostasis via the modulation of hepatic gluconeogenesis [54]. Fermented dairy product intake was associated with a lower risk of developing diabetes by the influence on intestinal microbiota and, thereby, on the insulin sensitivity of tissues and glucose tolerance [55]. Probiotics contained in such products may determine their favorable influence on T2DM risk [56]. A study by Liu et al. [50] revealed that the risk of T2DM was lower by 18% in women who consumed at least two servings of yoghurt weekly compared to women who consumed yoghurt less frequently than once a month (RR 0.82, 95% CI: 0.70–0.97, *p* = 0.03). Similar results were obtained in a study by Buziau et al. [57], in which the risk of developing T2DM was 19% lower in women from the highest tertile of yoghurt consumption than in women from the lowest tertile (OR 0.81; 95% CI: 0.67; 0.99; *p* = 0.041). It is consistent with the results obtained by Rosenberg et al. [58] and Margolis et al. [51] who confirmed a lower risk of developing T2DM in women consuming yoghurt. Detailed results of studies on the effects of dairy consumption on the risk of T2DM in women are described in Table 2.

Therefore, high dairy intake seems to reduce the risk of developing prediabetes and type 2 diabetes in women. It appears particularly beneficial to introduce yoghurt, fermented and low-fat dairy products into the diet. However, based on previous study results it may not be clearly confirmed whether the consumption of high-fat dairy products by women increased the risk of T2DM and had a negative impact on glycemia. Furthermore, due to the paucity of studies concerning the relationship between the consumption of milk products and the risk of T2DM in women with PCOS it seems justified to conduct a randomized clinical study in such a group of women in order to provide an explicit answer concerning the question of the influence of milk products on the course and treatment of PCOS.

## 4. Dairy Products versus Ovulation and Fertility in Women

Research on the influence of dairy products on female fertility and ovulation has been conducted for many years. The results of animal studies suggested a potentially unfavorable influence of dairy products on reproductive functions due to high lactose content, which reduced ovulation in rats and led to premature ovarian insufficiency. Moreover, it was demonstrated that rats fed with high amounts of galactose were characterized by markedly lower concentrations of estradiol and elevated levels of FSH (follicle stimulating hormone) and LH (luteinizing hormone). Rats fed with lactose had considerably reduced progesterone concentrations, while no differences were confirmed in serum estradiol concentrations [59,60].

Changes in hormone levels resulting from dairy product intake were also observed in studies conducted in people. According to Kim et al. [61] each increase in the consumption of dairy products by one serving per day was associated with the reduction in serum estradiol concentrations by 4.6% and free estradiol by 4.0%. Conversely, the highest total dairy intake was linked to an increase in LH concentrations by 2.9% over the whole cycle compared to the lowest intake. However, a study by Greenlee et al. [62] revealed that dairy products supported female fertility, because the participants drinking over three glasses of milk daily were characterized by a 70% drop in the risk of infertility compared to women who did not drink milk at all. Wise et al. [63] compared the cohorts of women from Denmark and North America. In both groups they observed a positive association between milk consumption and fertility. Moreover, the authors found no significant differences between low- and full-fat milk consumption as regards the influence on fertility in either of the cohorts. Furthermore, higher lactose intake was associated with higher fertility in the study cohorts, which is inconsistent with the previous accepted view stating that lactose impaired fertility. Additionally, Afeiche et al. [64] conducted a study on women undergoing assisted reproductive technology procedures. It was demonstrated that the group of women aged ≥35 who were in the highest quartile of dairy product intake prior to the treatment was characterized by a considerably higher probability of delivering a live neonate than women in the lowest quartile. Notably, the fat content of dairy products consumed by the participants had no influence on the strength of such a relationship. Contradictory results were arrived at by Souter et al. [65], who assessed the relationship between milk protein intake and antral follicle count (AFC) in a prospective group of women of reproductive age. They concluded that higher total milk protein intake (≥5.24% of energy value or ≥2.3 glasses of milk daily) was associated with lower AFC. The authors deduced that the factors influencing the reduction in antral follicle count in women consuming dairy products might include: high amounts of steroid hormones and growth factors present in dairy products, contamination of dairy products with pesticides and chemical substances, which might markedly affect endocrine function and folliculogenesis. Furthermore, an increased dairy intake may be associated with higher concentrations of IGF-I (insulin-like growth factor I) in the blood, which also produces a negative effect on ovarian function and antral follicle count.

A prospective cohort Nurses’ Health Study II (NHS II) did not reveal an association between total dairy intake and ovulatory infertility. However, increasing the consumption of low-fat milk products by one serving daily was linked to an increase in the risk of ovulatory infertility by 11%, while adding one serving of whole milk without increasing energy content was associated with reducing the risk by over 50%. According to the authors, it was due to the fact that high-fat dairy products included more estrogens and contributed to a lower-grade increase in IGF-I concentration in the serum compared to low-fat products. Moreover, based on the results, the authors hypothesized that the relationship between low-fat and full-fat dairy intake and infertility due to anovulation was stronger in women without certain clinical signs of PCOS than in women with those signs [66]. Notably, Adebamowo et al. [67] demonstrated that the consumption of skimmed milk was associated with a more common occurrence of acne, one of the clinical signs of PCOS, which may be explained by the presence of androgen precursors in milk. Rajaeieh et al. [68] studied the relationship between dairy products intake and the risk of developing polycystic ovary syndrome. They observed that each increase in milk consumption by one serving daily resulted in an increase in PCOS risk. Furthermore, women with this medical condition were characterized by a markedly higher consumption of low-fat or skimmed milk compared to healthy women. The authors noted a possible role in the pathogenesis of PCOS, because low-fat dairy products are characterized by a considerably higher strength of stimulating IGF-I secretion compared to full-fat products. Considering the low quality of evidence, it may not be explicitly concluded that the influence of dairy products on the risk of infertility and PCOS is unfavorable. Therefore, further research is necessary in this area [69].

## 5. Conclusions

It seems justified to include milk and dairy products into the diet of women with polycystic ovary syndrome because of the beneficial effect of those products on the risk of developing type 2 diabetes mellitus in women. Moreover, the products appear not to have a negative effect on ovulation and fertility in women. However, due to the lack of unambiguous evidence, the advantage of full-fat over low-fat dairy products may not be confirmed despite the fact that high-fat dairy intake seems to be more beneficial in polycystic ovary syndrome. Notably, studies concerning the influence of milk consumption in women with PCOS are scarce, so its beneficial effect may not be explicitly confirmed in this group of patients. Therefore, it is necessary to conduct well-designed extensive research in women with PCOS to lead to the final conclusion as to whether milk product consumption is beneficial in their case and which products should be selected: full-fat or skimmed ones.

## Figures and Tables

**Table 1:** The influence of dairy product consumption on insulin resistance in women.

Author/Reference Number	Year	Study Design	Sample (*n*)	Outcome Measures	Result
Intake of Total Dairy Products					
Pereira et al. [24]	2002	Population-based prospective study Intake of total dairy products	3157 Black and White adults aged 18 to 30 years	Fasting plasma insulin and glucose	An increase in the daily intake of milk products reduced the risk of insulin resistance by 30% in black women (OR 0.70, 95% CI, 0.54–0.91, *p* < 0.05) and by 38% in white women (OR 0.62, 95% CI, 0.46–0.84, *p* < 0.05).
Tucker et al. [19]	2015	Cross-sectional study Intake of total dairy products; low intake—0 to 0.5 servings of dairy per day, moderate intake—0.6 to 1.5 servings of dairy per day, high intake—1.6 to 6 servings of dairy per day	272 middle-aged, nondiabetic and apparently healthy women	HOMA-IR score	Women who consumed diet high in dairy products had markedly higher HOMA-IR values (0.41 ± 0.53) compared to those who consumed moderate (0.22 ± 0.55) and low amounts of dairy (0.19 ± 0.58).
Intake of Low-Fat Dairy Products					
Turner et al. [22]	2015	Randomized crossover study Intake of low-fat dairy products or red meat	47 overweight and obese men and women > 20 years old	Fasting insulin, HOMA-IR score, Matsuda Index	Fasting insulin was significantly higher after a diet including milk products compared to diet including red meat (7.38 versus 5.62, *p* = 0.02). HOMA-IR was significantly higher after a diet including low-fat milk products compared to diet including red meat (1.71 versus 1.31 *p* = 0.01). Insulin sensitivity calculated with the Matsuda method was lower by 14.7% in women who had a diet including milk products compared to diet including red meat (6.81 versus 8.14, *p* = 0.01). Women who consumed <1 portion of milk products daily were characterized by significantly lower fasting insulin levels and HOMA-IR, and a significantly higher Matsuda index compared to women whose diet included from 4 to 6 portions of low-fat milk products daily (fasting insulin—6.16 versus 7.38, *p* = 0.05, HOMA-IR—1.42 versus 1.71, *p* = 0.05 and Matsuda Index 8.61 versus 6.81, *p* = 0.05).
Intake of Milk and Milk Protein					
Lawlor et al. [18]	2005	Prospective cohort study Intake of milk versus no intake of milk	4024 British women aged 60–79 years	HOMA-IR score	Women who did not drink milk had their HOMA-IR lower by 13% compared to women who drank milk (1.49 versus 1.72).
von Post-Skagegård et al. [21]	2006	A randomized study Intake of three meals with different types of protein (either cod protein, milk protein or soy protein)	17 healthy women, 30–65 years old	Blood glucose, serum insulin, C-peptide	The 120 min insulin to glucose ratio was higher after a meal including milk protein compared to meals including cod or soy protein (milk protein—4.36, cod protein—2.03, soy protein—2.78, *p* = 0.0002). The 120 min insulin to peptide C ratio was significantly higher in case of a meal including milk protein compared to meals including cod or soy protein (milk protein—0.008, cod protein—0.003, soy protein—0.005, *p* = 0.001).
Drouin-Chartier et al. [23]	2015	Randomized, crossover study, diet for 6 weeks, one with 3.2 servings/d of 2% fat milk per 2000 kcal and another without milk	27 postmenopausal women in good health with abdominal obesity, less than 70 years of age	Fasting glucose, fasting insulin, Matsuda Index	No effect of milk on fasting insulin levels and insulin sensitivity index. Both diets, with and without milk, significantly reduced fasting glucose levels (diet including milk—6.08 versus 5.77, *p* < 0.001, diet not including milk—5.98 versus 5.80, *p* < 0.009).
Intake of Yoghurt					
Madjd et al. [28]	2016	Randomized single-blind controlled trial Intake of low-fat yoghurt versus probiotic yoghurt	Overweight and obese women	Fasting plasma glucose, 2 h glucose, fasting plasma insulin, HOMA-IR score, HbA1c	A significantly higher reduction was observed as regards HOMA-IR, 2 h postprandial glucose and fasting insulin in a group of women consuming probiotic yoghurt. Fasting glucose levels, 2 h glucose level, HbA1c, fasting insulin and HOMA-IR significantly decreased in both groups.
Jafari et al. [27]	2016	Randomized, placebo-controlled, double-blind parallel-group clinical trial Intake of vitamin D fortified yoghurt versus plain low-fat yoghurt for 12 weeks	59 post-menopausal women with type 2 diabetes	HOMA-IR, QUICKI	Insulin sensitivity of tissues was increased in a group of women who consumed yoghurt fortified with vitamin D—HOMA-IR (3.32 versus 2.13, *p* = 0.02), QUICKI (0.331 versus 0.348, *p* = 0.001) and fasting insulin was reduced (7.71 versus 5.17, *p* = 0.03). The markers of carbohydrate metabolism deteriorated in a group of women consuming low-fat yoghurt.
No Dairy Products in the Diet					
Phy et al. [20]	2015	Intervention study 8-week diet without starch and dairy products	24 overweight and obese women (BMI ≥ 25 kg/m^2^ and ≤ 45 kg/m^2^) with PCOS	Fasting and 2 h glucose and insulin, HOMA-IR score	Diet without starch and milk products reduced fasting insulin by 52% (−17.0 ± 13.6 μg/mL, *p* < 0.001), 2 h insulin in the load test of 75 g glucose by 37% (−82.8 ± 177.7 μg/mL, *p* = 0.03) and HOMA-IR by 51% (−1.9 ± 1.2, *p* < 0.001).

HOMA-IR, Homeostatic Model Assessment—Insulin Resistance; QUICKI, Quantitative Insulin Sensitivity Check Index; BMI, body mass index; HbA1c, glycated hemoglobin; PCOS, polycystic ovary syndrome; OR, odds ratio; CI, confidence interval.

**Table 2:** The influence of dairy product intake on the risk of type 2 diabetes mellitus (T2DM) in women.

Author/Reference Number	Year	Study Design	Sample (*n*)	Outcome Measures	Results
Pittas et al. [48]	2006	Prospective cohort study Intake of total dairy products	83,779 apparently healthy women, aged 30–55 years	T2DM	The risk of T2DM lower by 13% in women consuming higher amounts (>3 servings/day) of dairy products compared to women consuming small amounts (<1 serving/day).
Liu et al. [50]	2006	Prospective cohort study Intake of total dairy products, low-fat, full-fat and yoghurt	37,183 healthy, middle-aged and older women	T2DM	The risk of T2DM lower by 20% in women consuming higher amounts (>2.9 servings/day) of dairy products compared to women consuming small amounts (<0.85 serving/day). The risk of T2DM lower by 18% in women consuming higher amounts (>2 servings/day) of low-fat dairy products compared to women consuming small amounts (≤0.27 serving/day). The risk of T2DM lower by 18% in women consuming higher amounts (>2 servings/week) of yoghurt compared to women consuming small amounts (<1 serving/month).
van Dam et al. [52]	2006	Prospective cohort study Intake of total, low-fat and full-fat dairy products	41,186 women, aged 21–69	T2DM	The risk of T2DM lower by 25% in women consuming higher amounts (>2 servings/day) of total dairy products compared to women consuming small amounts (<1 serving/week). The risk of T2DM lower by 13% in women consuming higher amounts (>1 servings/day) of low-fat dairy products compared to women consuming small amounts (<1 serving/week). No significant correlation between the risk of T2DM and the consumption of full-fat dairy products in women.
Kirri et al. [49]	2009	Prospective cohort study Intake of total dairy products, milk, cheese and yoghurt	33,919 middle-aged and older women	T2DM	The risk of T2DM lower by 29% in women consuming higher amounts (≥300 g/day) of dairy products compared to women consuming small amounts (<50 g/day). No correlation between the consumption of milk, cheese and yoghurt and the risk of T2DM in women.
Malik et al. [53]	2011	Prospective cohort study	116,671 female registered nurses aged 24–42	T2DM	The risk of T2DM lower by 25% in women from the highest quintile of total milk product consumption compared to women from the lowest quintile. The risk of T2DM lower by 26% in women from the highest quintile of low-fat milk product consumption compared to women from the lowest quintile. The risk of T2DM lower by 28% in women from the highest quintile of high-fat milk product consumption compared to women from the lowest quintile.
Margolis et al. [51]	2011	Prospective cohort study Intake of total, low-fat, full-fat dairy products and yoghurt	82,076 women, aged 50–79	T2DM	The risk of T2DM lower by 21% in women consuming higher amounts (>2.6 servings/day) of total dairy products compared to women consuming small amounts (<0.7 serving/day). The risk of T2DM lower by 30% in women consuming higher amounts (>1.9 servings/day) of low-fat dairy products compared to women consuming small amounts (<0.2 serving/day). The risk of T2DM lower by 54% in women consuming higher amounts (≥2 servings/week) of yoghurt compared to women consuming small amounts (<1 serving/month).
Aune et al. [34]	2013	Systematic review and dose-response meta-analysis of cohort studies Intake of total, low-fat, full-fat dairy products, milk and yoghurt	526,482 healthy men and women ≥ 20 years	T2DM	The risk of T2DM in women reduced by 34% with the increase in milk consumption by 200 g daily. The risk of T2DM in women reduced by 33% with the increase in yoghurt consumption by 200 g daily. No significant correlation with the total, full-fat, low-fat milk product consumption and cheese consumption in women.
Gijsbers et al. [32]	2016	A dose–response meta-analysis of observational studies Intake of total dairy products	579,832 healthy men and women, aged ≥ 20 years	T2DM	The risk of T2DM decreased by 3% with the increase in total dairy intake by 200 g daily. The risk of T2DM in women decreased by 8% with the increase in low-fat dairy intake by 200 g daily. The risk of T2DM in women increased by 2% with the increase in low-fat milk consumption by 200 g daily. The risk of T2DM in women reduced by 5% with the increase in high-fat milk consumption by 200 g daily. The risk of T2DM in women reduced by 11% with the increase in yoghurt consumption by 50 g daily. No correlation between the risk of T2DM and the total, high-fat milk product consumption and cheese consumption in women.
Mishali et al. [35]	2019	Systematic review and meta-analysis of prospective cohort studies with subgroup analysis of men versus women Intake of total dairy products	545,677 men and women aged ≥ 18 years	T2DM	The risk of T2DM lower by 13% in women consuming higher amounts of dairy products compared to women consuming small amounts.
Buziau et al. [57]	2019	Prospective cohort study Intake of yoghurt	8748 Australian women, aged 45–50	T2DM	The risk of T2DM lower by 19% in women from the highest tertile of yoghurt consumption compared to women from the lowest tertile.
Rosenberg et al. [58]	2020	Prospective cohort studyT otal intake of yoghurt	59,000 U.S. Black women, aged 21–69	T2DM	The risk of T2DM lower by 18% in women consuming higher amounts (≥1 serving/day) of yoghurt compared to women consuming small amounts (<1 serving/month).
